# Microfluidic chip and isothermal amplification technologies for the detection of pathogenic nucleic acid

**DOI:** 10.1186/s13036-022-00312-w

**Published:** 2022-12-01

**Authors:** Dongli Gao, Xudong Guo, Yi Yang, Hua Shi, Rongzhang Hao, Shengqi Wang, Zhen Jun Li, Rongtao Zhao, Hongbin Song

**Affiliations:** 1 College of Public Health, Zhengzhou University, Zhengzhou, Henan China; 2 Chinese PLA Center for Disease Control and Prevention, Beijing, China; 3 Beijing Institute of Microbiology and Epidemiology, Beijing, China; 4 State Key Laboratory for Infectious Disease Prevention and Control, National Institute for Communicable Disease Control and Prevention, Chinese Center for Disease Control and Prevention, Beijing, China

**Keywords:** Isothermal amplification, Microfluidic chip, Point-of-care testing, Recombinant polymerase isothermal amplification

## Abstract

The frequency of outbreaks of newly emerging infectious diseases has increased in recent years. The coronavirus disease 2019 (COVID-19) outbreak in late 2019 has caused a global pandemic, seriously endangering human health and social stability. Rapid detection of infectious disease pathogens is a key prerequisite for the early screening of cases and the reduction in transmission risk. Fluorescence quantitative polymerase chain reaction (qPCR) is currently the most commonly used pathogen detection method, but this method has high requirements in terms of operating staff, instrumentation, venues, and so forth. As a result, its application in the settings such as poorly conditioned communities and grassroots has been limited, and the detection needs of the first-line field cannot be met. The development of point-of-care testing (POCT) technology is of great practical significance for preventing and controlling infectious diseases. Isothermal amplification technology has advantages such as mild reaction conditions and low instrument dependence. It has a promising prospect in the development of POCT, combined with the advantages of high integration and portability of microfluidic chip technology. This study summarized the principles of several representative isothermal amplification techniques, as well as their advantages and disadvantages. Particularly, it reviewed the research progress on microfluidic chip–based recombinase polymerase isothermal amplification technology and highlighted future prospects.

## Background

In the last decades, the epidemic events of newly emerging infectious diseases, such as atypical pneumonia, influenza, Ebola hemorrhagic fever, and Middle East respiratory syndrome, have frequently occurred, posing a serious threat to human health and causing a heavy burden on the public health and economic development of societies [1]. China faces an unprecedented threat in terms of the prevention and control of imported newly emerging infectious diseases as well as biosecurity pressure with the accelerated globalization of the world economy and more frequent flow of people [2, 3]. Infectious diseases can spread through various routes. Among these, respiratory infectious diseases are more likely to cause an outbreak of the epidemic due to their fast transmission speed, easily realized transmission mode, and short latency. The “five early” measures of early detection, early diagnosis, early reporting, early quarantine, and early treatment are the key to the epidemic prevention and control of infectious diseases. Since the outbreak of COVID-19 at the end of 2019, as of May 1, 2022, the cumulative number of confirmed COVID-19 cases in the world has exceeded 500 million [4]. The rapid spread of the epidemic around the world makes it particularly important to develop rapid and accurate real-time testing methods for severe acute respiratory syndrome coronavirus (SARS-CoV-2). Rapid and accurate identification of infectious agents is an important prerequisite for scientific and precise implementation of the “five early” measurements, as well as a key strategy for the early implementation of intervention measures and prevention of infectious disease outbreaks [5–7].

Conventional pathogen detection methods mainly include the culture-based method, immunological assays, molecular biological assays, and so forth. Among these, the culture-based method is considered the gold standard for pathogen detection. However, it is inefficient and reliant on a professional operation. Thus, this method fails to meet the needs of pathogen detection for a sudden outbreak of the epidemic. Immunological assays are limited in their wide application because of their relatively low sensitivity [8]. The development of nucleic acid detection technology, as represented by PCR, has led to revolutionary progress in detecting pathogenic microorganisms. Compared to the culture method, PCR has the advantages of high efficiency and high sensitivity. Hence, it has become a commonly used method in the field of molecular diagnosis and is widely applied in several fields, including the diagnosis of infectious diseases, screening of animal epidemics, and food safety [9]. PCR can be used to detect pathogenic microorganisms with high sensitivity and high specificity. Particularly, precise quantification of target detection can be achieved via digital PCR [10–12]. Quantitative real-time fluorescent PCR (qRT-PCR) is currently the most commonly used method for detecting infectious disease pathogens. However, this assay requires cumbersome sample pretreatment, nucleic acid extraction, and purification, as well as complex thermal cycling processes, while it is reliant on expensive detection equipment and professional laboratory conditions. Hence, qRT-PCR fails to meet the needs of on-site real-time detection of infectious diseases such as COVID-19. Given the current high prevalence of the COVID-19 epidemic, the laboratory-based standardized PCR detection methods have been unable to meet the current needs of testing volume and timeliness. On March 11, 2022, China issued the “Notice on Printing and Distributing COVID-19 Antigen Detection Application Plan (Trial)” [13], in which antigen testing was recommended as a supplement to nucleic acid testing. Antigen testing is easier to popularize and relatively inexpensive. However, compared with nucleic acid testing, it displays a low detection accuracy that can easily lead to missed detection.

Point-of-care testing (POCT) refers to the diagnostic tests performed outside the central laboratory infrastructure near the location of patients, generating a rapid test result using portable analytical instruments and supporting reagents on site. In recent years, rapid progress has been made in the development of POCT worldwide. POCT may serve as the main method for detecting and diagnosing infectious diseases in resource-poor countries or regions [14, 15]. POCT can shorten the detection time and provide a timely diagnosis and treatment for patients with acute and those in remote areas, thus, reducing morbidity and mortality [16]. Miniaturization, portability, and easy operation of the analytical system are the main features of POCT. At present, POCT still has some problems in clinical and on-site application. Compared with the conventional laboratory testing techniques, POCT needs to be optimized and has not yet realized standardized quality control, operation, and result reporting. The testing cost of POCT is relatively high [17, 18], and a gap exists in the stability and reliability of testing between POCT and qRT-PCR. The growing maturity of the integrated and automated microfluidic chip detection system, as well as the wide application of nucleic acid isothermal amplification techniques characterized by rapidity and constant temperature has provided an opportunity for developing a more portable, sensitive, reliable, and low-cost POCT.

## Main text

### Microfluidic chip technology

Microfluidic chip technology can be used to integrate some or even all of the process units required for nucleic acid detection, such as sample pretreatment, nucleic acid extraction, target sequence amplification, and signal detection, into one single micron-scale chip [19]. Compared with the traditional laboratory-developed nucleic acid detection methods, this technology possesses advantages, including high integration, compactness, portability, less sample consumption, easy operation, and so forth. Hence, it has been widely used in various fields such as molecular biology, chemical analysis, clinical medicine, and food hygiene [20–22]. The continuous development and maturity of microfluidic chip technology can help solve the puzzles involving the separation of sample pretreatment, nucleic acid amplification, and detection steps as well as cumbersome operations, realize the automatic integration of nucleic acid detection, increase the detection efficiency, minimize operating errors, and reduce the detection costs and risks of aerosol contamination. Microfluidic technology has remarkably expanded the application field of *in vitro* diagnosis. More importantly, it provides technical support for the development of POCT devices applicable for various test settings.

The completion of the entire reaction process in microfluidic chips is dependent on the fluid driving system. Currently, the fluid driving of microfluidic chips is realized mainly through automatic or manual means [23–25].

**Fig. 1:**
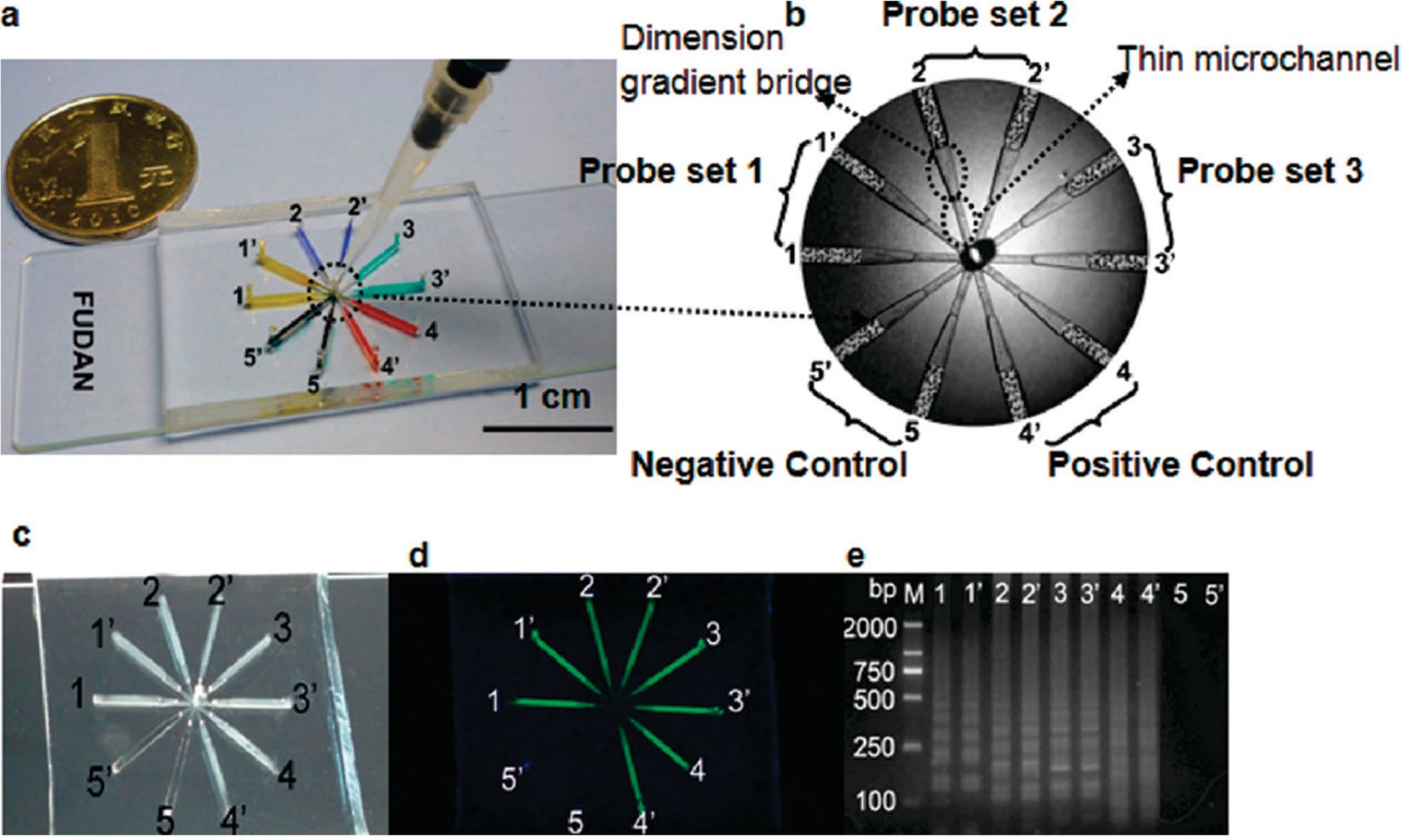
MμLAMP for the point-of-care analysis of multiple genes. **a** Photograph of the 10 microchamber mμLAMP system in a PDMS-glass format. **b** Structure of the mμLAMP chip. Microchambers were connected to the corresponding thin microchannel via dimension gradient bridges with the whole shape like an octopus. **c** Direct naked-eye determination of the assay result via the insoluble byproduct magnesium pyrophosphate. **d** These results were further validated by the green fluorescence induced by the DNA-intercalating dye SYBR green I and (**e**) the characteristic ladderlike pattern of the LAMP product determined by agarose gel electrophoresis [27]

**Fig. 2:**
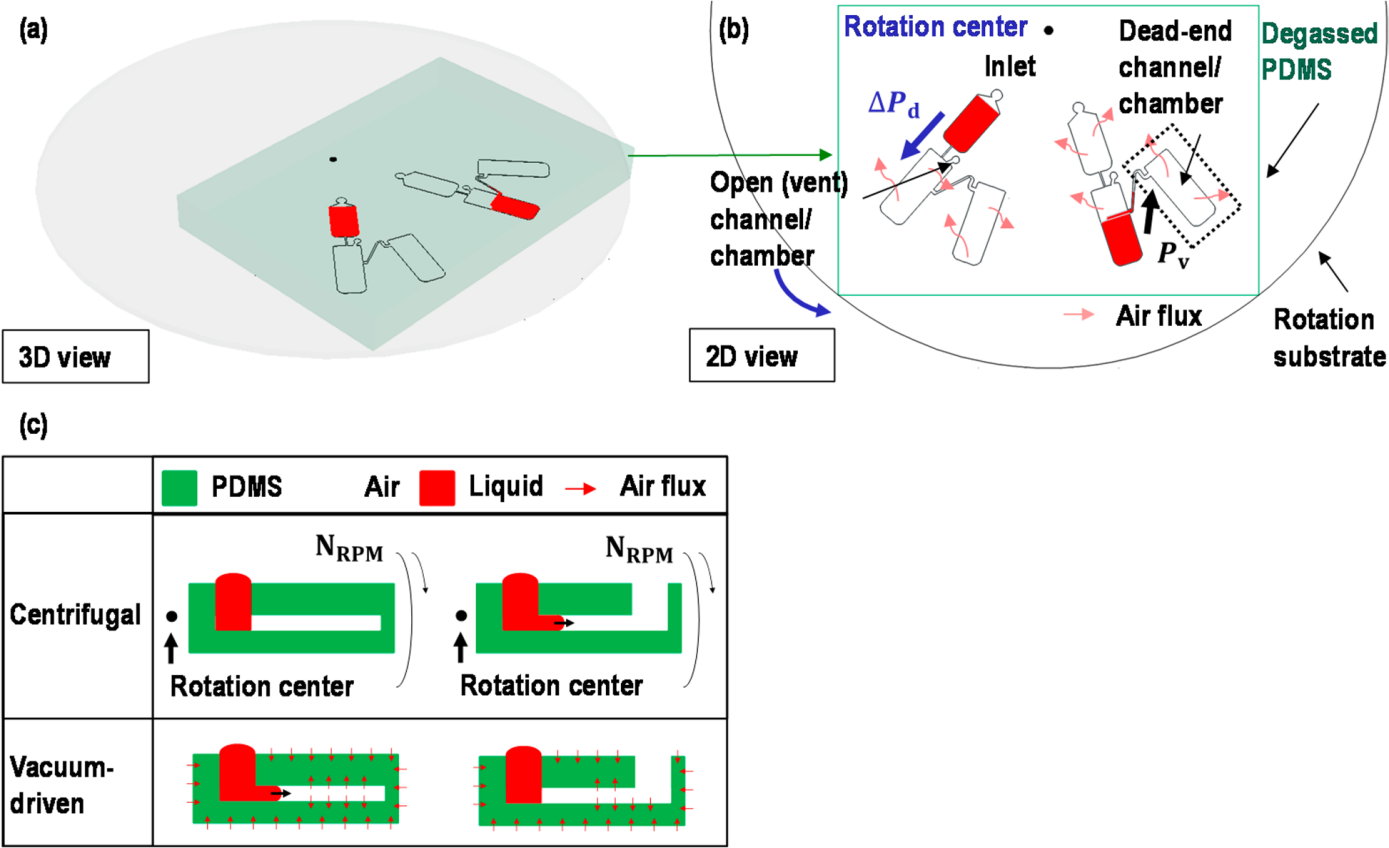
Schematic diagram of the microfluidic device. **a** 3D and (**b**) 2D schematics of the operation principle using driving pressure by centrifugal force, ΔP_d_, or vacuum-driven pressure, P_v_. The centrifugal pressure allowed the sample to be driven in a one-way direction, while the vacuum-driven force could be used in various directions to drag the sample without any rotation. **c** Comparison between the centrifugal flow and the vacuum-driven flow in each dead-end and open-end channel. The centrifugal pressure required an open-end channel to drive a sample, while the vacuum-driven pressure required a dead-end channel to drive a sample [29]

Automation-driven microfluidic devices employ capillary force, vacuum-driven force, or chemical reaction–generated gas to distribute the reaction solution into the microchannels. In this case, the most common lateral flow chromatography test strips use the capillary force to drive the flow of reagents [26]. Fang Xueen et al .[27] developed a multiplex microfluidic system based on loop-mediated isothermal amplification (mμLAMP). For the assay, both the sample and LAMP reaction buffer were added to the central well and subsequently filled into all 10 reaction microchambers via capillary action. This system was capable of qualitatively and quantitatively analyzing the three subtypes of influenza A viruses (Fig. 1). In 2017, Renner developed a vacuum-driven microfluidic chip called B-chip for detecting *Enterococcus faecium, S. aureus, Klebsiella pneumoniae, Acinetobacter baumannii, Pseudomonas aeruginosa, and Enterobacter spp* (ESKAPE) pathogens [28]. This detection system comprised an inexpensive heater, a light-emitting diode, a fluorescent filter, and a plastic box. For the assay, the primers, probes, and enzymes were pre-packed into 16 detection wells, and the gas was discharged by vacuuming. Upon loading the samples, the reaction mixture was distributed into each well under the action of vacuum suction, while the excess liquid flowed into the waste liquid pool. This chip system dramatically simplified the liquid processing procedure using vacuum-driven fluid flow. Wang et al. [29] developed a centrifugal force– and vacuum-driven microfluidic device using gas-soluble polydimethylsiloxane (PDMS) as the device material (Fig. 2). The multi-directional transport of samples could be achieved with this device. Thus, the one-way transport of samples in centrifugal force–driven devices is no longer an unsolved problem. This device used centrifugal force to transport liquid samples into the chambers. Once pre-degassed PDMS material was exposed again to the air, it automatically absorbed the air in the internal microfluidic channel. As a result, a vacuum was formed in the channel to drive the sample liquid flow. Upon loading the reagents, the auto-driven microfluidic device automatically controlled the flow rate and quantity while no additional operation was needed. Hence, this system provided great convenience to users. However, it still had several limitations, such as a slow flow rate and long analysis time. Moreover, the auto-driven microfluidic device could hardly meet the requirements for the multi-directional transport of liquid because pressure could only be generated in one direction. All these weaknesses limited the scope of its application.

**Fig. 3:**
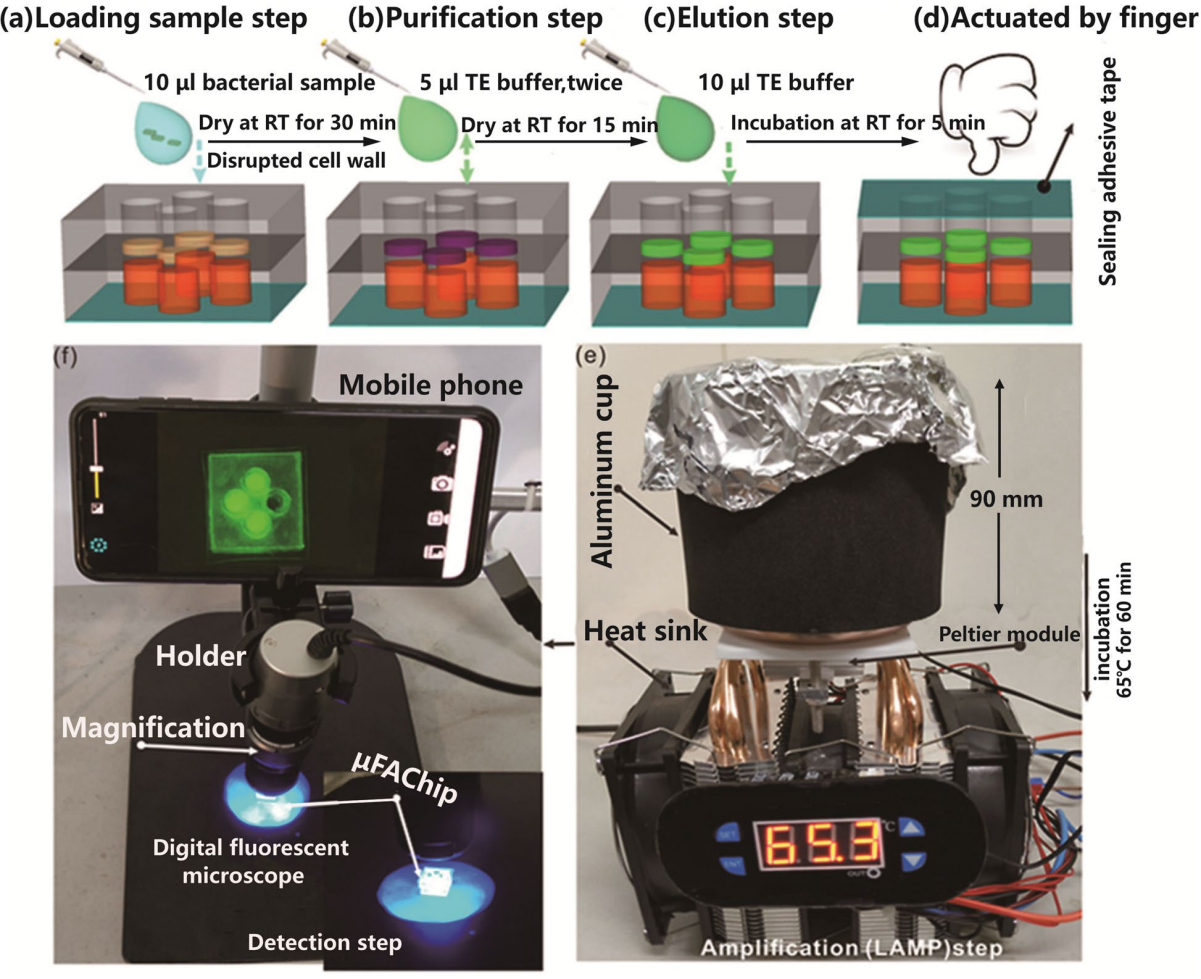
Images depicting integrated and finger-actuated microfluidic chip for point-of-care testing of multiple pathogens. **a**-**d** The schematic illustration of the overall experimental process including DNA extraction, amplification and detection. **e** A photograph of the custom-built peltier heater. **f** The portable fluorescent imaging system [30]

**Fig. 4:**
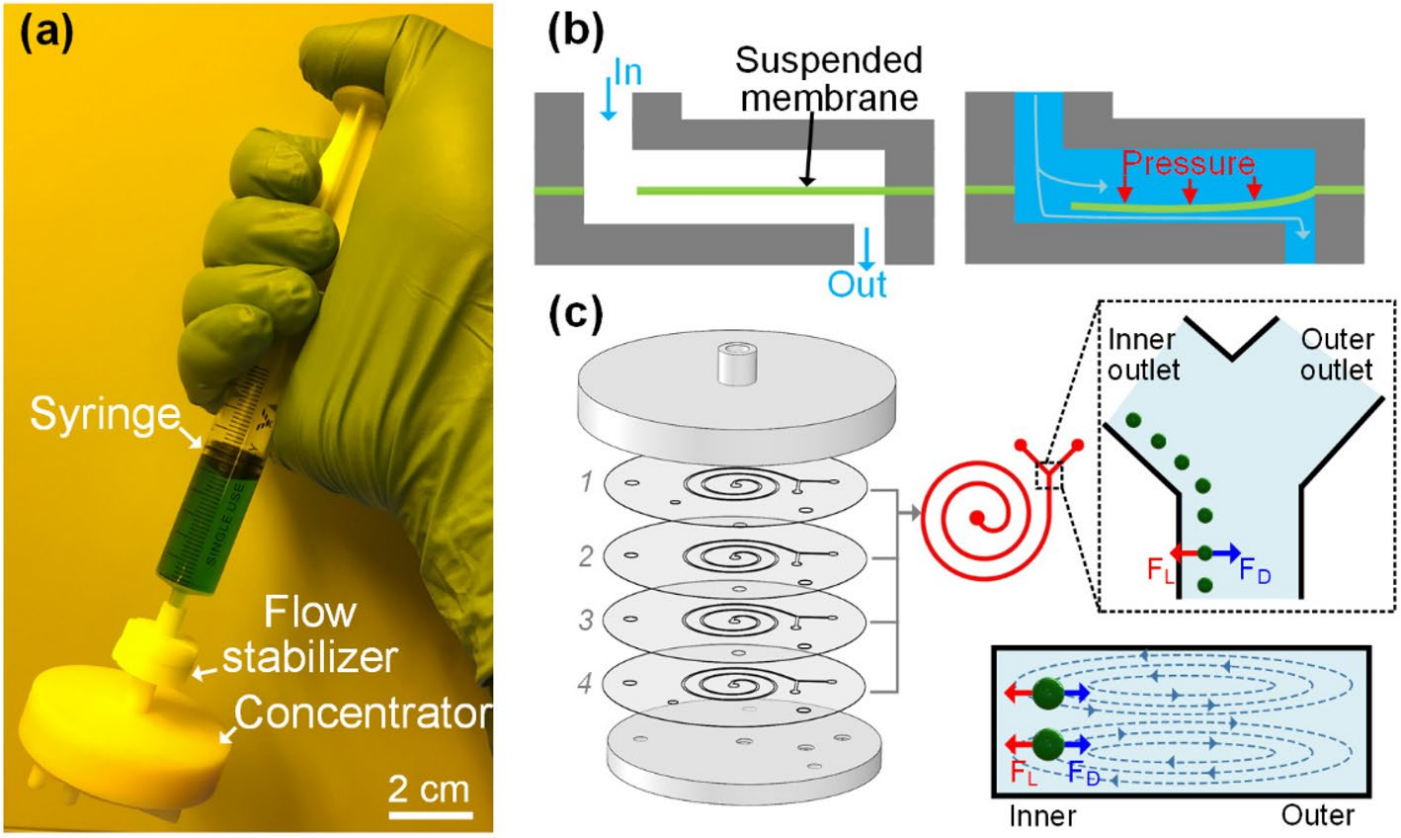
**a** Photograph illustrating the operation of the hand-operated syringe-tip inertial microfluidic centrifuge (i-centrifuge) for continuous-flow cell concentration. **b** Working principle of the flow stabilizer for regulating varied input liquid flow to be at a desired stable flow rate. **c** Structure and working principle of the syringe filter-like inertial microfluidic concentrator [31]

Manually driven microfluidic devices use human power to achieve fluid actuation via simple devices such as syringes, pipettes, and finger presses. Chen et al. [30] developed a finger-actuated microfluidic chip that could be used to simultaneously detect different types of bacterial pathogens. Upon loading the reagents, the valve was opened by simply pressing the finger on the top layer of the chip and the nucleic acid samples in the upper chamber subsequently flowed into the reaction chamber in the bottom layer for a reaction (Fig. 3). Xiang et al. [31] reported a hand-operated and syringe-driven microfluidic centrifuge called i-centrifuge (Fig. 4). This centrifuge was composed of a syringe-tip flow stabilizer and a four-channel parallel inertial microfluidic concentrator. Also, it had several advantages, such as low equipment cost, simple manual operation, high flow-rate processing, portable equipment size, and single use. Compared with the automatically actuated system, the manually actuated microfluidic device could apply variable pressures to the microfluidic channel, thus being conducive to the fluid control in the microchannel and capable of controlling liquid flow and direction in a more diversified manner. However, the connection between the manually actuated device and other components of the microfluidic system might complicate the whole system. Moreover, this system was inferior to the automatically actuated microfluidic chip in terms of automatic operation and precise liquid control.

### Isothermal amplification techniques

Isothermal nucleic acid amplification technique is an *in vitro* amplification method for rapidly amplifying nucleic acids at a constant temperature using enzymes with distinct functions and specific primers. Since the early 1990s, various isothermal amplification techniques have been developed one after another. Compared with PCR, isothermal amplification has the advantages of simplicity, rapidity, and high efficiency, and does not require complicated thermal cycling equipment, thus, significantly reducing the requirements for the experimental environment and hardware conditions. Therefore, many scholars believe that isothermal amplification may potentially serve as an alternative detection technique to PCR. At present, the commonly used isothermal amplification methods mainly include LAMP, nucleic acid sequence–based amplification (NASBA), recombinase polymerase amplification (RPA), rolling circle amplification (RCA), helicase-dependent amplification (HAD), and so forth.

### Loop-mediated isothermal amplification

LAMP was first reported by Notomi et al. in 200 0[32]. For the amplification, four to six primers need to be designed. At 60–65°C, a strand-displacing *Bst* DNA polymerase catalyzes the primer extension along the template and the strand displacement reaction for 1 h, ultimately giving rise to the target repeat fragments of different lengths. The amplified products can be detected using ELISA [33], gel electrophoresis, real-time turbidimetry, lateral flow test strips [34, 35], and fluorescent probe method [36, 37]. LAMP has become a research hotspot for the development of POCT in the last 20 years because of its advantages such as high sensitivity (two to five orders of magnitude higher than the traditional PCR assays), low instrument requirements, and judgment of the test results based on the visual observation of white turbidity or generated green fluorescence. However, LAMP has some disadvantages. First, false-positive results could be a serious problem due to aerosol pollution during amplification [34]. Second, it is difficult to design appropriate primers for the gene sequences of some pathogens because of relatively high requirements for primer design. Third, the final product usually comprises fragments of different lengths, thereby limiting its downstream application [38]. Thus, these disadvantages impact the promotion and practical application of LAMP to a large extent.

### Nucleic acid sequence–based amplification

NASBA is an *in vitro* nucleic acid amplification technique reported by Compton in 1991 [39]. For the amplification, the template is preheated at 65°C for 5 min to remove the secondary structure of RNA, and the amplification reaction is then conducted at a constant temperature of 42°C in a standard reaction system. The first primer serves as the initial reverse-transcription primer, which simultaneously serves to add a T7 promoter to the resulting cDNA. After cDNA synthesis, the RNA in the newly formed heteroduplex is degraded by RNase H allowing the second DNA primer to hybridize, resulting in a dsDNA after extension by reverse transcriptase (RT). This dsDNA can then be transcribed by T7 RNA polymerase. As the resulting RNA can in turn be reverse-transcribed, exponential amplification occurs [40] . The amplified products can be detected mainly by electrophoresis, ELISA [41], or the molecular beacon method. NASBA has the advantages of high specificity and sensitivity. Moreover, the samples are not easily contaminated during the amplification, and the detection of RNA viruses is not susceptible to interference from DNA in the template. Hence, it can be used to detect RNA viruses such as respiratory viruses [42, 43] and hepatitis A viruses [44]. Nevertheless, NASBA is a relatively high-cost method. Besides, the technology is mostly in the research stage, and a true constant temperature for the amplification is not realized due to the requirement of preheating treatment prior to the testing.

### Recombinase polymerase amplification

RPA is an isothermal amplification technique established by Piepenburget et al. in 2006 [45], which involves recombinase T4 uvsX, polymerase Bsu, single strand-binding protein gp32, and two primers. Once the protein–DNA complex formed by the binding of recombinase to the primer via the homologous sequence in the double-stranded DNA, the recombinase catalyzes the DNA strand exchange reaction. Subsequently, the primer-directed DNA synthesis is initiated by the polymerase, and exponential amplification of the target region on the template occurs. Meanwhile, the replaced DNA strand binds to single strand DNA-binding to prevent further replacement. Moreover, the thermal denaturation of the template is not required for RPA, and the amplified product can be obtained within 30 min at 37–42°C. The results of RPA can be assessed using a number of assays, including agarose gel electrophoresis, real-time detection of fluorescent probes [46], and lateral flow chromatography [47]. RPA has become the fastest-developing isothermal amplification technique in recent years due to its rapidity, high sensitivity, and mild reaction conditions at a constant temperature. It has some limitations such as relatively long primer probes, difficulty in designing the primers, and restrictions on the storage of enzymes. However, it is considered the most promising alternative option to PCR as a key member of isothermal nucleic acid amplification techniques [48].

### Rolling circle amplification

RCA is an isothermal amplification reaction catalyzed by a DNA polymerase with strand displacement activity at a constant temperature of 37°C [49]. A padlock probe is hybridized with the target sequence through the action of DNA ligase and then ligated into a circular template. Upon being aligned with the circular template, the primer is extended along the loop through the action of DNA polymerase, and the previously generated extension chains are constantly replaced. Ultimately, repetitive long single-stranded DNA products are generated. RCA can be applied to the detection of pathogenic microorganisms [50, 51], with the advantages of single reaction primers and high specificity. However, the DNA ligase used for the circular reaction in RCA has relatively high requirements on the reaction system. Also, the technique is cumbersome and unable to meet the needs of inexpensive detection due to its high cost. Moreover, a rapid detection cannot be achieved because of several hours of reaction time, while the padlock probe and DNA template in the reaction system produce strong background signals that can affect the detection limit. In fact, as an isothermal amplification technique, RCA needs to be optimized in various aspects such as probe design, background elimination, cost, and time [52].

### Helicase-dependent amplification

Helicase-dependent amplification (HAD) is a helicase-based isothermal nucleic acid amplification technique developed by New England Biolabs in the USA [53]. This technique mimicks the *in vivo* DNA replication process at a constant temperature of 65°C *in vitro*. Single-stranded DNAs are generated from double-stranded DNAs by DNA helicase. Two specific primers, P1 and P2, bind to the respective targets to form a partial DNA duplex and are extended through the action of DNA polymerase. As a template, the newly synthesized DNA duplex is unwound by a thermostable helicase and then enters into the next round of amplification reaction, thereby fulfilling DNA amplification under isothermal conditions. HDA can only be applied to the amplification of short-length DNA fragments. Also, it has some disadvantages such as low amplification rate and time-consuming process (60–120 min), which limit its practical application in POCT.

In the last 20 years, continuous progress has been made in developing various isothermal nucleic acid amplification techniques and signal detection strategies for more sensitive, simple, and rapid detection of pathogenic nucleic acids. In this study, we comparatively analyzed the amplification time, temperature, advantages, and disadvantages among several main isothermal amplification techniques (Table 1).

**Table 1:** Comparative analysis of various isothermal amplification methods

Amplification methods	LAMP	NASBA	RCA	HDA	RPA/RAA
Template	DNA and RNA	RNA	DNA and RNA	DNA and RNA	DNA and RNA
Reaction temperature	65°C	37-42°C	37°C	65°C	37-42°C
Amplification time	60 min	90 min	30-240 min	60-120 min	10-30 min
Enzymes for amplification	*Bst* DNA polymerase	Reverse transcriptase, RNase H, and T7 RNA polymerase	DNA ligase and DNA polymerase	Helicase, single-stranded DNA-binding protein, and DNA polymerase	Recombinase, *Bst* DNA polymerase, and single-stranded DNA-binding protein
Number of primers	4-6	2	1	2	2
Detection methods for the amplified products	Double-stranded chimeric dye, turbidimetry, indicator lateral flow chromatography, gel electrophoresis, and ELISA	Molecular beacon probes, gel electrophoresis, and ELISA	Fluorescent probes and gel electrophoresis	Gel electrophoresis and fluorescent probes	Fluorescent probes, lateral flow chromatography, and gel electrophoresis
Advantages	Simple reaction system, multiple detection methods, and product detection with naked eyes	Direct detection of RNAs and prevention of contamination	High specificity and low risk of contamination	Low and constant temperature and high sensitivity	Rapid detection, low and constant temperature
Disadvantages	False positivity, complex primer design, and being prone to nonspecific amplification	A necessary preheating step and failure in achieving a real constant temperature	More operating procedures and longer reaction duration	Longer reaction duration	Longer primer probes and being easy to form dimers

### Pathogen detection techniques based on RPA in combination with microfluidic chips

Integration of isothermal amplification technology into microfluidic chips or portable devices greatly improves nucleic acid–based on-site pathogen detection, thus, facilitating the development of POCT diagnosis based on nucleic acid detection [54]. A comprehensive comparison of technical characteristics among several isothermal amplification methods reveals that RPA combined with microfluidic chips has huge potential in developing more portable and mature POCT techniques due to its advantages of short detection time and low and constant temperature. The techniques for pathogenic microorganism detection based on RPA in combination with microfluidic chips have been widely studied and applied in various fields, including medicine, food industry, and molecular biology.

**Fig. 5:**
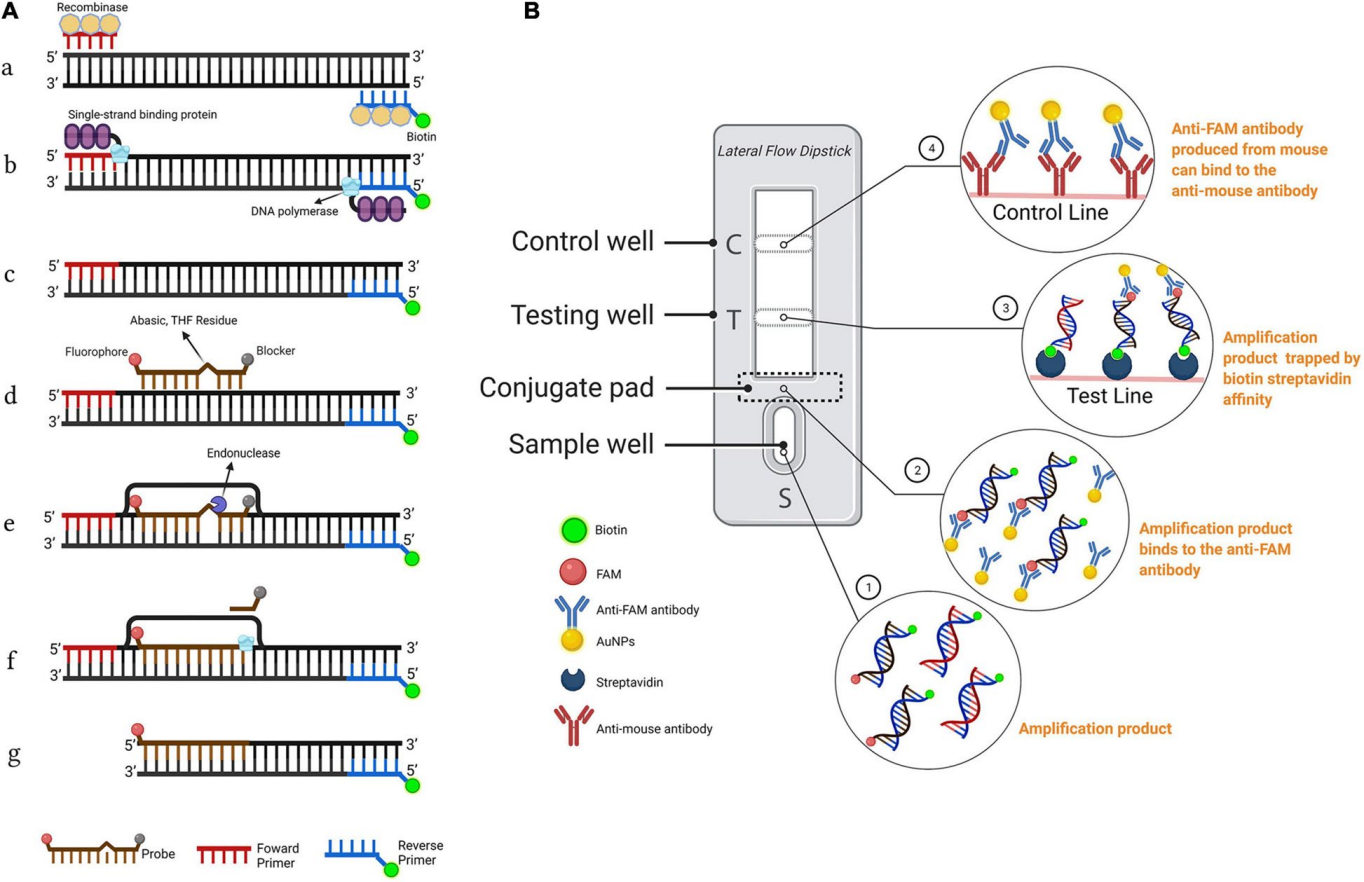
Schematic representation of the recombinase polymerase amplification combined with lateral flow dipstick (RPA-LFD) assay. **A** Principle of RPA. **B** Schematic representation of the LFD working principle [55]

The lateral flow dipstick (LFD) assay is a relatively common portable detection technique categorized into microfluidic chips in a broad sense. RPA combined with lateral flow (LF) test strips possesses the advantages of rapidity and portability in the POCT and has been widely studied. Zhou et al. [55] developed an on-site detection method RPA-LFD for *Bursaphelenchus xylophilus* based on RPA in combination with LF test strips (Fig. 5). The assay can be completed in around 30 min at 38°C. Notably, the detection limit can be as low as 1 pg of purified *B. xylophilus* genomic DNA, and the assay displays a high specificity. Although RPA-LFD assay has the advantages of short detection time, low instrument dependence, and suitability for on-site testing, it is limited by a relatively high detection cost (approximately 50 yuan/test, which is higher than that for PCR assay). Zhao et al. [56] developed a method for specifically detecting *Mycobacterium bovis* DNA using RPA combined with LFD. The successful detection of *M. bovis* DNA with this assay can be achieved within 30 min at 39°C. Strikingly, this assay displays a detection limit of 20 copies/μL as well as a sensitivity four times as high as that of real-time quantitative PCR (qPCR). Sandeep et al. [57] established an RPA-LFD assay for detecting *Mycoplasma ovipneumoniae*, which helped obtain visual test results within 20 min at 39°C. In particular, this assay showed a detection limit of 9 copies/reaction, comparable to the sensitivity of real-time fluorescence quantitative PCR, while it had a higher specificity. The direct exposure of the test strips to the environment during the assay procedure could easily lead to a risk of aerosol contamination. In fact, this problem could be effectively solved by sealing the test strip into the microfluidic chip.

**Fig. 6:**
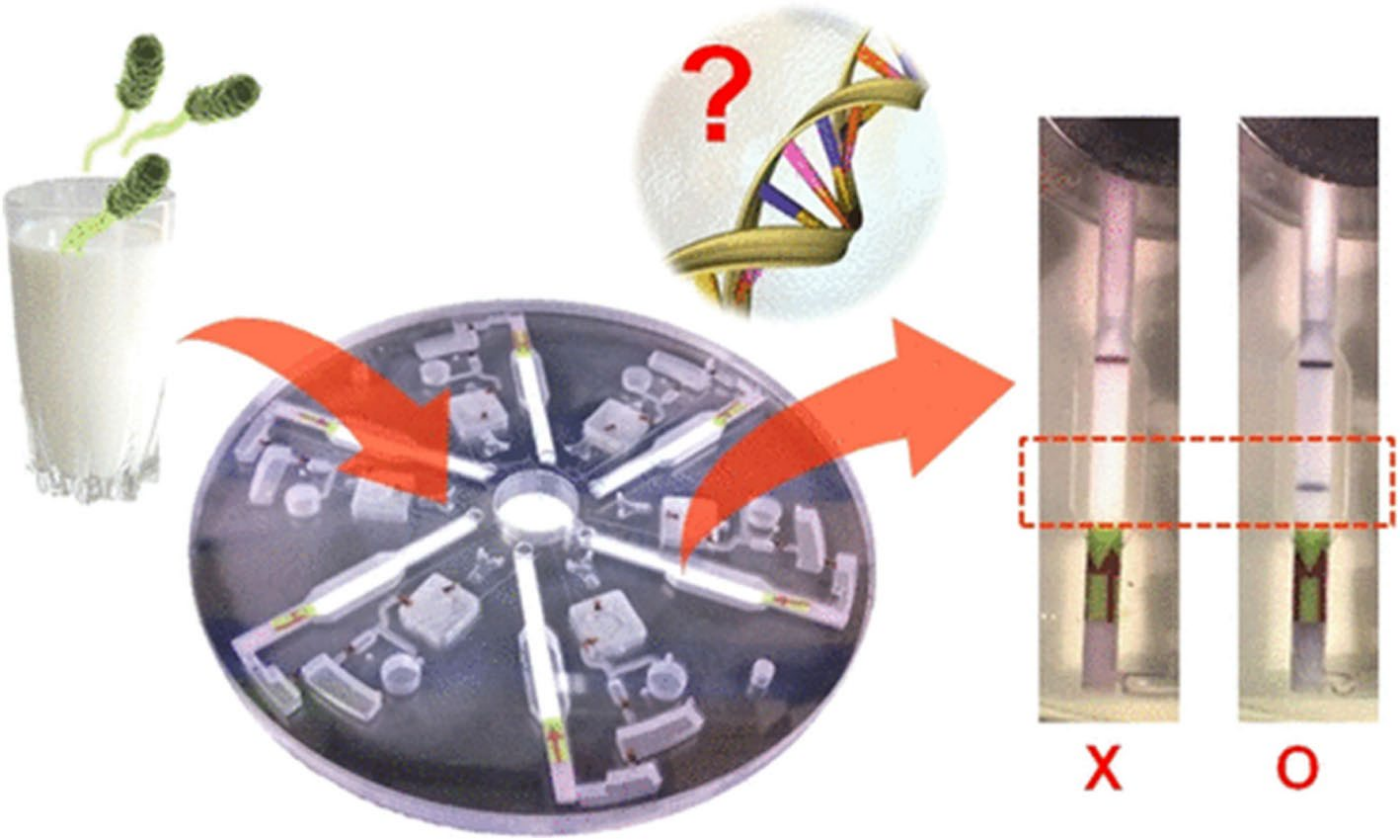
Schematic diagram of RPA and disk chip combination. The chip integrates nucleic acid extraction and amplification, and the results are detected using the integrated side flow chromatography strip [58]

In 2014, Kim [58] developed a centrifugal microfluidic assay device by integrating RPA, LF test strips, and a disk microfluidic chip (Fig. 6). In this device, the three main steps, including pathogen nucleic acid extraction, DNA amplification, and visualization of the results, are integrated on one single chip. The assay can be completed within 27.5 min. Notably, this assay displays a detection limit of 10 CFU/mL or 10^2^ CFU/mL for *Salmonella* in phosphate-buffered saline or milk, and the test results can be directly interpreted using the test strips integrated in the chip. This device possesses the advantages of high integration of multiple detection steps, which greatly simplify the test procedure. Meanwhile, the entire procedure is carried out in a sealed chip. As a result, a possible contamination caused by the opening of the test tube during the traditional test strip assay can be effectively avoided. Given that this device is relatively complex in design and bulky, further improvement in portability needs to be done.

**Fig. 7:**
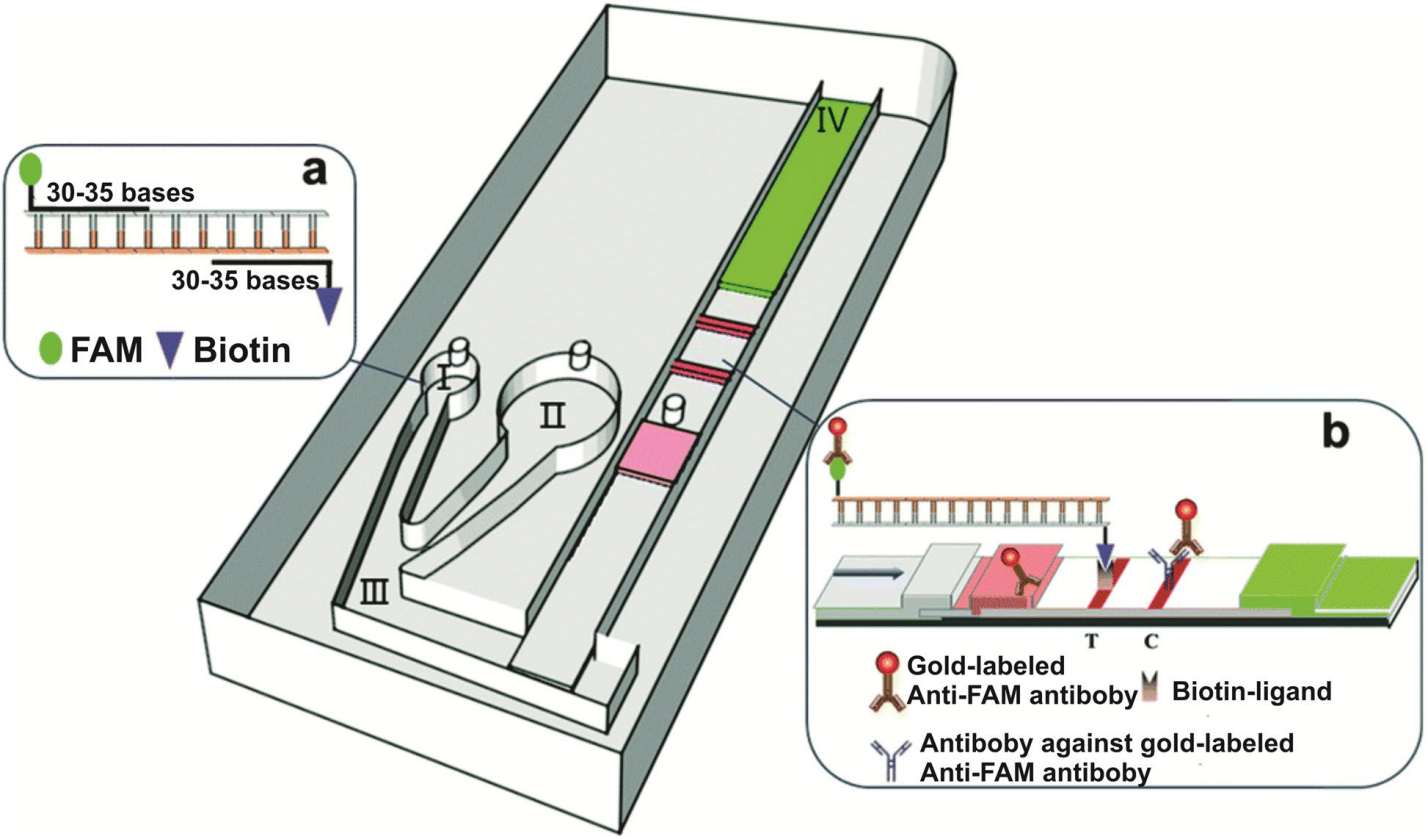
Schematic illustration of the MI-IF-RPA system for COVID-19 detection. Samples containing COVID-19 target genes are loaded into the small reservoir (I), where they are isothermally reverse transcribed, amplified, labeled with biotin and FAM, then bound to the anti-FAM antibody. The samples are then mixed (III) with running buffer from the large chamber (II) for capillary diffusion across detection bands in the lateral flow chamber (IV) [26]

Liu et al. developed an MI-IF-RPA assay for rapid COVID-19 detection by integrating reverse-transcription RPA (RT-RPA) and LF test strips on one single microfluidic chip [26] (Fig. 7). RT-RPA-amplified reaction components in chamber (I) are mixed with the running buffer of chamber (II) in chamber (III), and then delivered to the LF detection strips for biotin- and FAM-labeled amplified analyte sequences. Around 30 min later, the results can be interpreted with naked eyes. Strikingly, this assay displays a detection limit of 1 copy/μL or 30 copies/sample for SARS-CoV-2-armored RNA particles. The validation of clinical samples and comparison with RT-PCR revealed that the MI-IF-RPA assay had a sensitivity of 97% and a specificity of 100%. The enclosed design for the MI-IF-RPA assay can effectively prevent possible aerosol contamination. Compared with the centrifugal microfluidic assay device mentioned earlier, the MI-IF-RPA system has a significantly reduced instrument volume and improved portability. However, the nucleic acids must be extracted from the samples before the test. As a result, more operation steps are required for this assay, and its promotion and application have been limited to a certain extent.

**Fig. 8:**
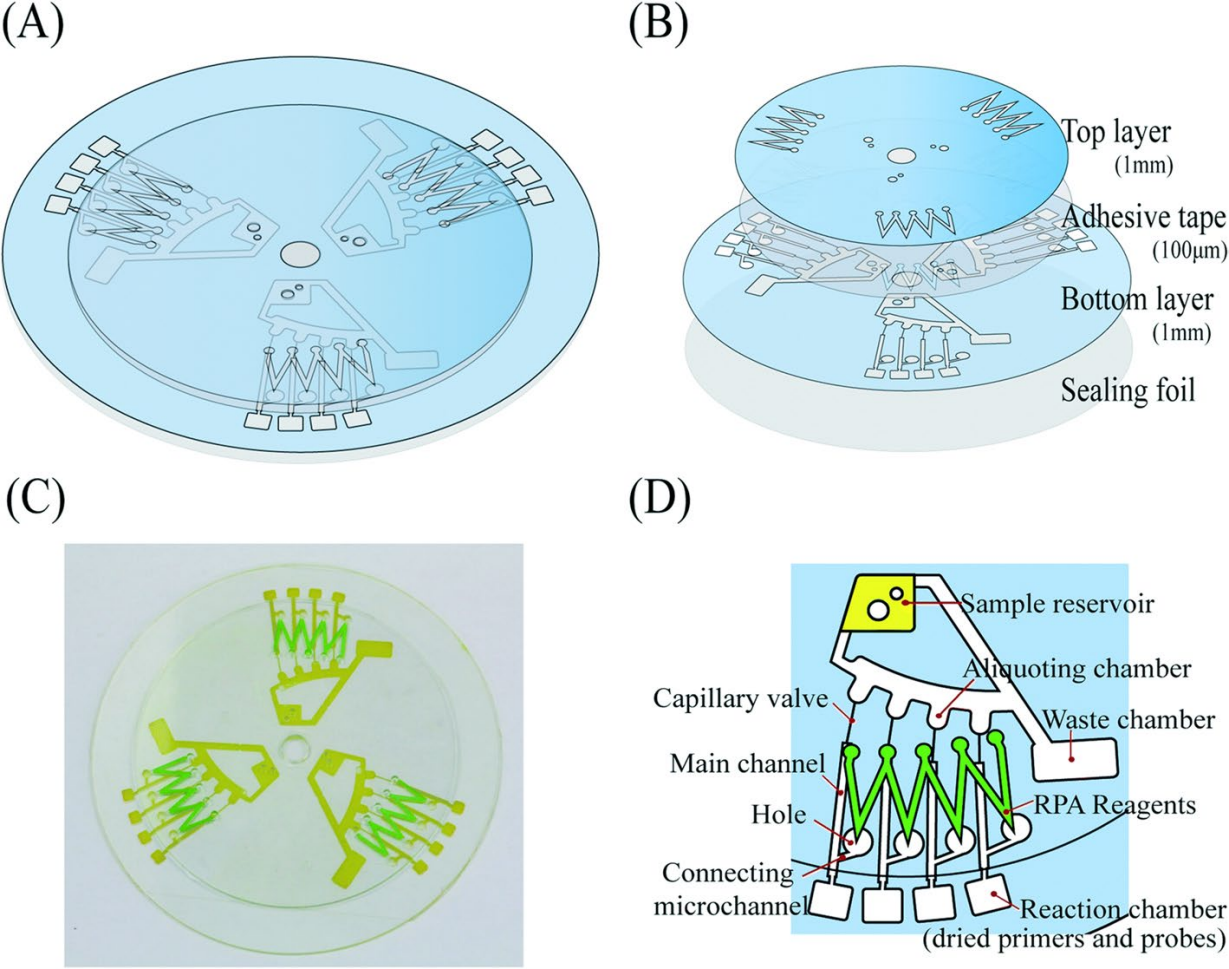
Schematic illustration of the centrifugal direct-RPA microdevice. **A** An assembled image, **B** a disassembled image, **C** a real digital image of the microdevice, and **D** an enlarged schematic of each unit and its components [59]

**Fig. 9:**
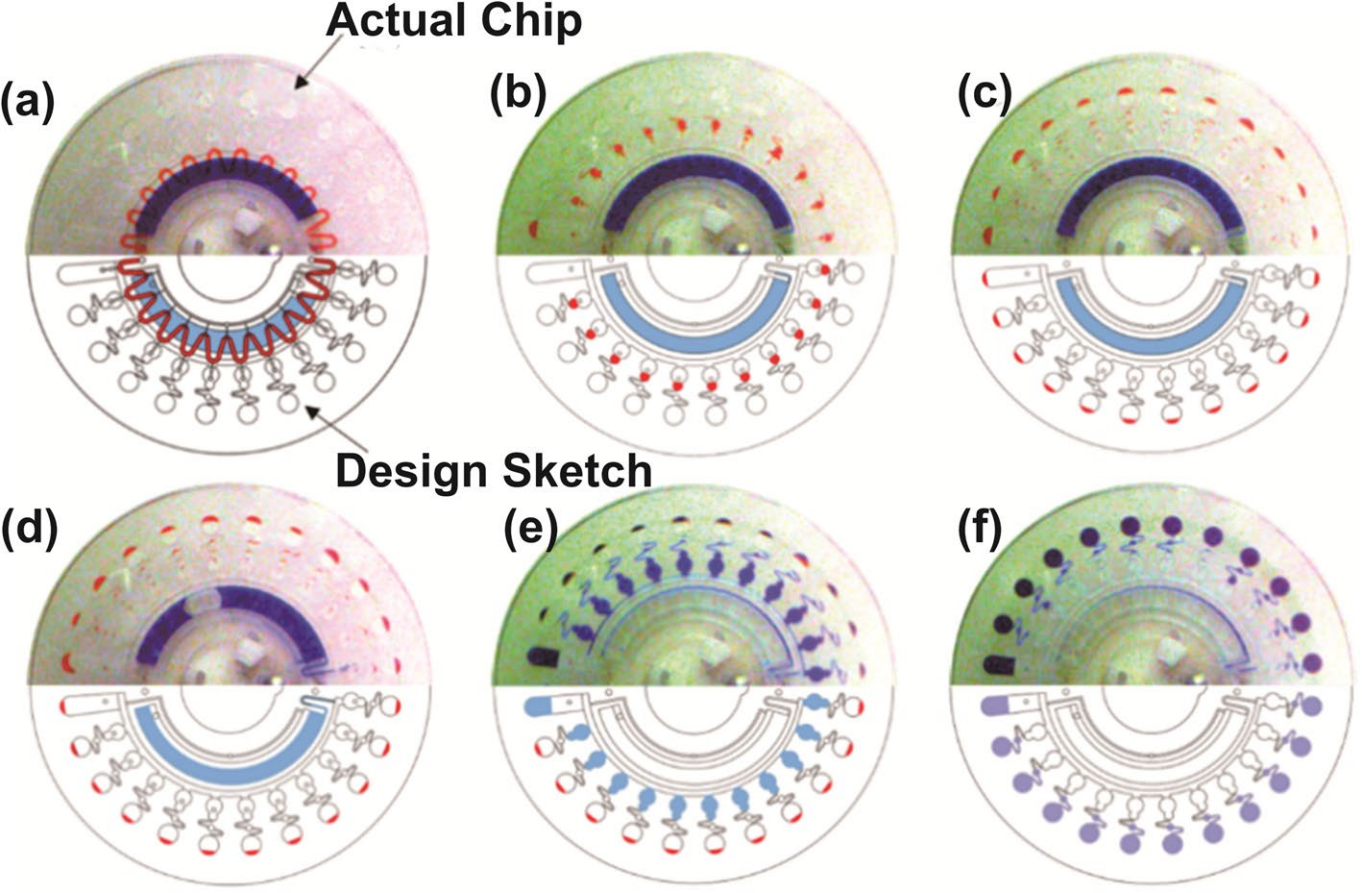
Illustration of the entire flow control of the chip. **a** Initial state of the chip with the RPA mix (red dye) and LAMP mix (blue dye) ,dried primers. **b** RPA mix was divided into quantitative chambers at 2000 rpm for 30 s. **c** RPA product was transferred into the amplification chamber as the template for LAMP. **d** LAMP mix was primed into the siphon valve by the capillary action at 100 rpm for 30 s. **e** LAMP mix was transferred into the separated sub-volumes at 2000 rpm for 30 s. **f** LAMP mix was distributed into reaction chambers at 6000 rpm for 60 s [60]

In 2016, Choi developed a centrifugal direct-RPA microdevice for the rapid detection of multiple foodborne pathogens in milk samples [59]. In this device, the flow direction and rate of the fluids can be modulated by designing channel configuration and adjusting the rotation speed to fulfill nucleic acid isolation, isothermal amplification, and fluorescence assay (Fig. 8). This microdevice consists of three identical functional units with four reaction chambers for each unit. Thus, 12 direct-RPA reactions can be performed simultaneously. Moreover, specific and simultaneous detection of multiple pathogens can be achieved on a single microdevice. For assaying milk samples, the sensitivity of this device can reach 4 cells/3.2 μL within 30 min. At present, this device can only be applied to detect Gram-negative bacteria because it is unable to disrupt the outer peptidoglycan layer of Gram-positive bacteria for nucleic acid extraction. Huang et al. developed a microfluidic chip detection system using two-stage isothermal amplification, including first-stage RPA and second-stage LAMP, which provided a simple molecular diagnostic method for the real-time and parallel detection of multiple targets in clinic, with a minimum detection limit of approximately 10 copies [60] (Fig. 9). In this case, the microfluidic chip–based two-stage isothermal amplification integrates the advantages of both RPA and LAMP, while avoiding their disadvantages. Thus, this system can be used to detect various types of nucleic acid samples, including DNA and RNA, in a simultaneous and timely manner. Strikingly, it requires fewer reagents and involves low cost and no cross-contamination, thereby facilitating the detection of clinical samples that may contain both bacteria and RNA viruses, as well as accurate classification for timely diagnosis and treatment. Besides, the flow and transfer of the samples can be achieved by adjusting the number of revolutions. However, this assay is time-consuming and usually takes 1 h to obtain the test results.

**Fig. 10:**
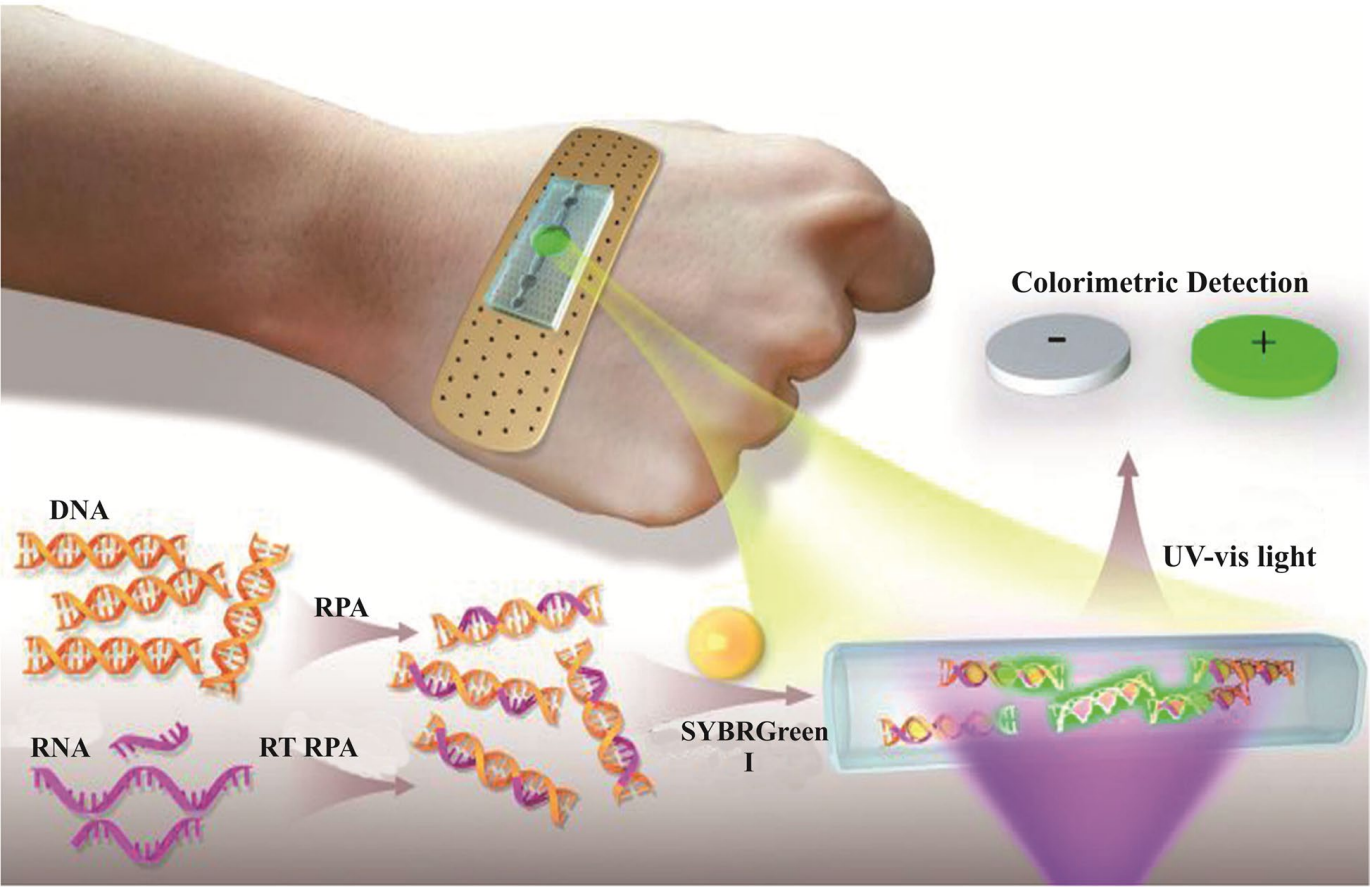
Schematic of the wearable microfluidic sensor for nucleic acids [61]

**Fig. 11:**
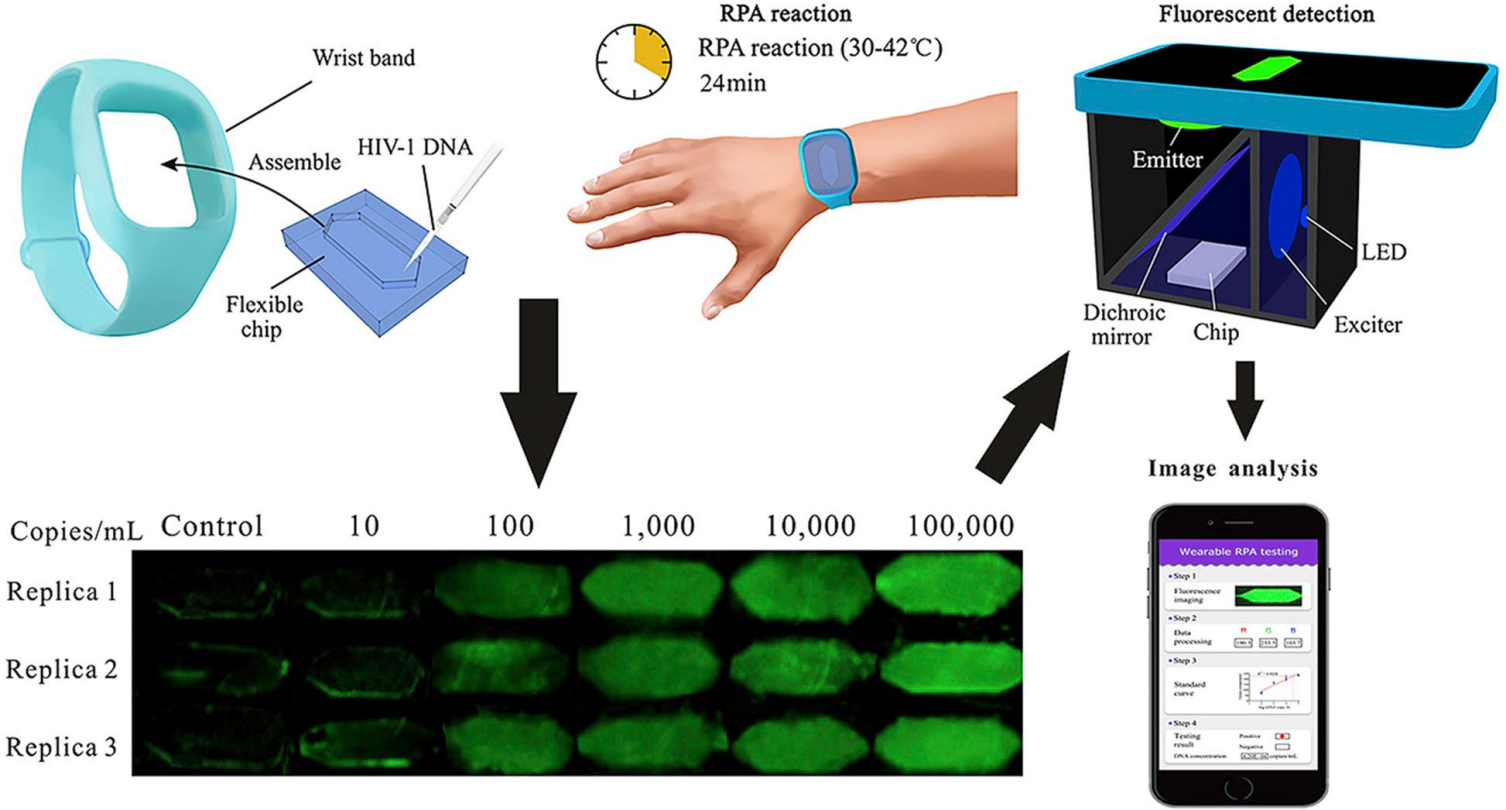
Schematic of wearable RPA testing for the rapid detection of HIV-1 DNA [62]

Wearable biosensors that can be directly worn or attached to the body surface or integrated into clothing and accessories are gradually popularized and applied due to their good portability, providing a new strategy for real-time and sustainable health monitoring. Yang et al. combined the flexible microfluidic technique with RPA and developed a novel bandage-type wearable microfluidic-RPA sensor for the rapid and intuitive detection of Zika virus nucleic acids [61] (Fig. 10). In the sensor, the human body temperature contributes to the required temperature for RPA reaction. The nucleic acid sample is incubated for 10 min, and the fluorescence is excited by irradiation with a mini ultraviolet flashlight. A smartphone can be used to record the images, and the test results are interpreted with naked eyes. In the meantime, Kong et al. developed a wearable microfluidic device based on human body temperature–triggered RPA reaction for simple and rapid amplification of HIV-1 DNA [62] (Fig. 11). The test results could be obtained within 24 min with the help of a mobile phone–based fluorescence detection system. The detection limit was 100 copies/ml. This wearable RPA-microfluidic detection device has the advantages of being fast, sensitive, and easy to use, thus, contributing to the development of POCT, especially the self-detection of pathogens. For assays using the two devices mentioned earlier, nucleic acid extraction from body fluid samples needs to be carried out prior to the testing. In these cases, the automatic and real-time detection of pathogens cannot be achieved.

**Fig. 12:**
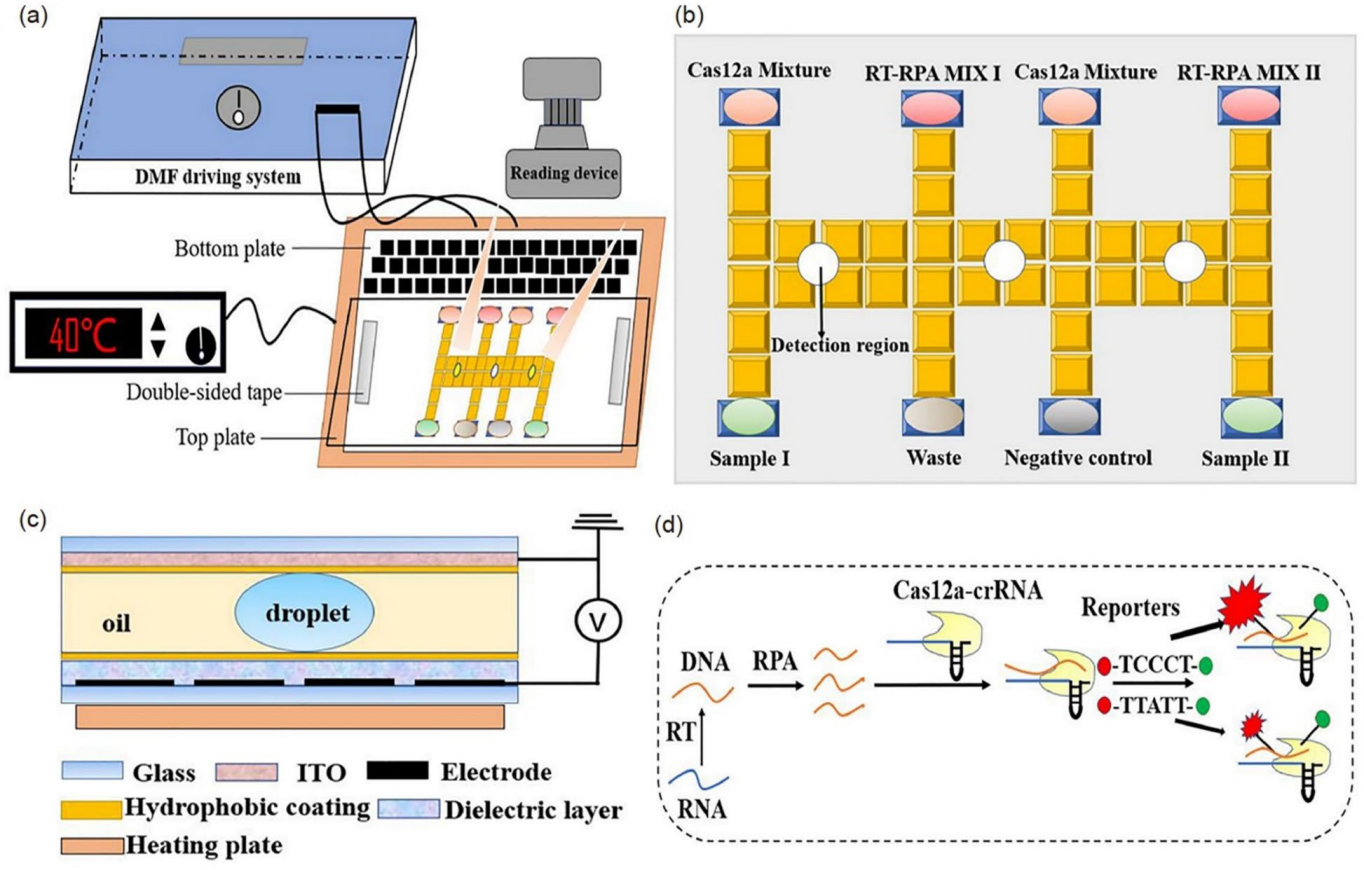
**a** Illustration of the RCD platform. **b** Plan view of the electrodes of the DMF chip and each reagent in the reservoir. **c** Side view of the DMF chip including a droplet. **d** Schematic illustration of the RPA-Cas12a-crRNA recognizing target and cleaving the reporter [63]

Digital microfluidics (DMF) is an emerging technology that can be employed for a complex laboratory assay based on precise droplet manipulation. DMF has broad application prospects in biology due to its advantages such as portability, reagent saving, and reduced power consumption. Sun et al. developed an automated nucleic acid detection platform (RCD) for influenza viruses and SARS-CoV-2, which was based on optimized RPA-Cas12a combined with DMF [63] (Fig. 12). The RCD platform uses DMF to control the reaction droplets at the microliter level, promoting the mixing efficiency of droplets. In the meantime, the RPA-Cas12a system can improve the amplification efficiency and amplify the fluorescence signals. In this case, the assay can be completed within 30 min, and the detection limit of target RNAs reaches 5.2 copies. It was reported that this platform provided consistent results with qPCR in assaying 52 clinical samples. Moreover, this platform could integrate multiple electrodes, thereby possessing an application potential in multi-channel detection. Although this platform provides a rapid and sensitive assay, the extraction and purification of nucleic acids prior to the test on the machine lead to an increase in the number of detection procedures.

**Fig. 13:**
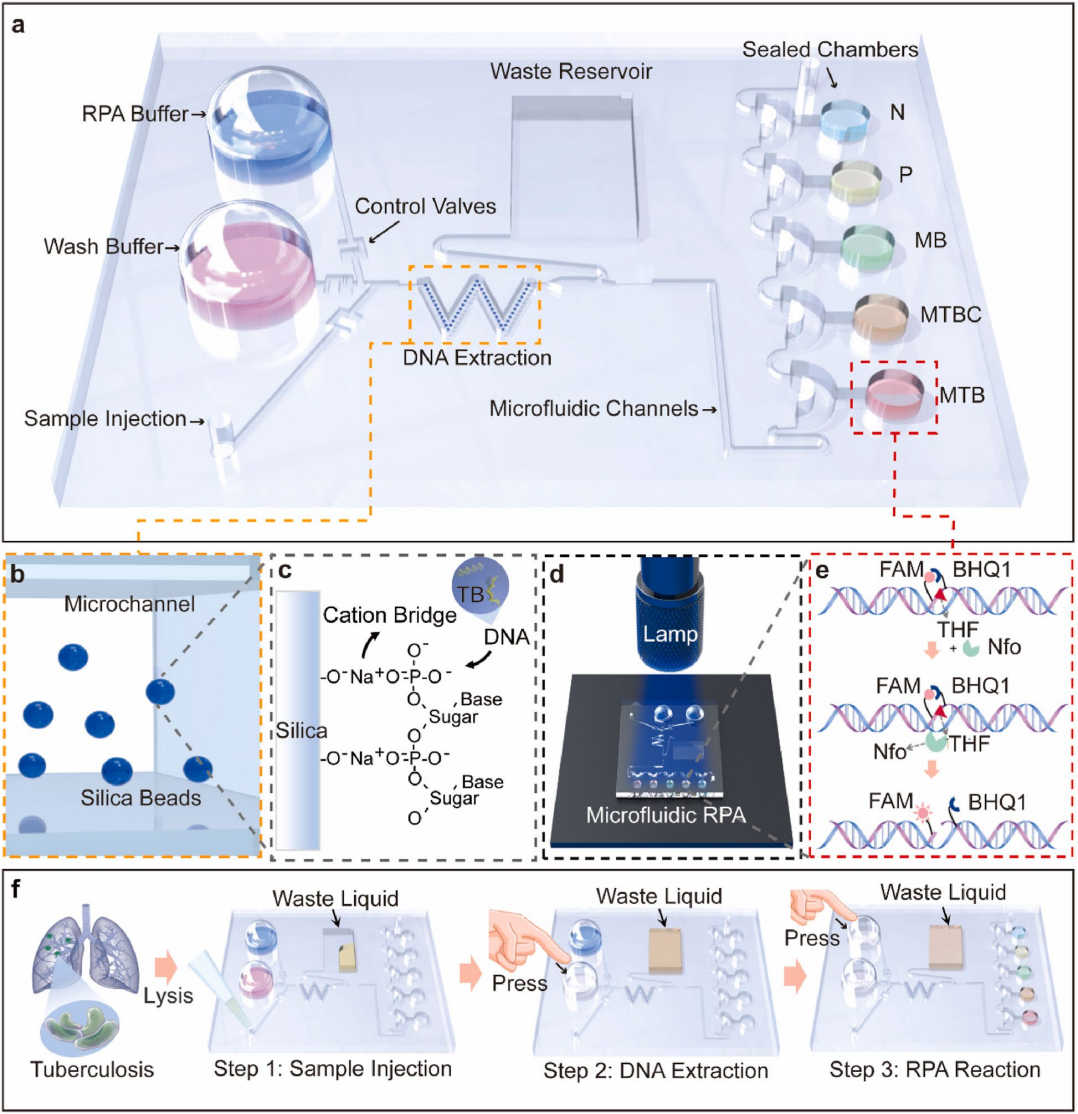
Fd-MC based on RPA for the rapid detection of tuberculosis (TB). **a** Overview of the Fd-MC capable of performing diverse functions, including DNA solid-phase extraction (SPE), RPA amplification, and multiplexed detection. **b** SPE unit was pre-filled with silica particles to capture the DNA of the pathogens in the sample. **c** DNA molecules of TB-family pathogens were adsorbed by the silica beads through a cationic salt bridge. **d** Fluorescence signal of the RPA amplicon was emitted by a hand-held blue lamp at an excitation wavelength of 480 nm for visual diagnosis. **e** Mechanism of fluorescence generation in the presence of the target DNA. **f** Operation steps for TB detection, from sample injection, DNA extraction to the final RPA amplification [64]

The development of POCT with sample-in-answer-out capability has an important application value for the early detection in resource-limited settings such as rural areas, remote districts, and outbreak sites of the epidemic. Still, a gap exists between the development of RPA- and microfluidic chip–based POCT technology and the realization of real on-site integrated rapid detection. Particularly, integrating high-quality nucleic acid extraction and amplification detection procedure on the microfluidic chip is currently the technical challenge to be addressed. In 2021, Wang et al. described a low-cost and portable RPA-based finger-driven microfluidic system (Fd-MC) for rapidly detecting tuberculosis (TB )[64] (Fig. 13). This system employs the pressure generated by finger pressing to drive the fluid flow. After pretreated liquid samples are injected, the nucleic acids are enriched in the W-shaped microchannel of the microfluidic chip using magnetic beads and then transported together with the RPA buffer to multiple fully isolated microchannels for the completion of the amplification procedure. Finally, the fluorescent probe is excited using a hand-held ultraviolet lamp, and the test results are interpreted. Fd-MC adopts a multi-channel detection design, enabling the simultaneous detection of multiple pathogens with a sensitivity of 10^3^copies/ml. It can not only identify TB/non-TB infection but also distinguish human *Mycobacterium* from *M. bovis*. A test of 37 clinical specimens revealed a specificity of 100% and a sensitivity of 95.2%. Moreover, this system integrates sample pretreatment and nucleic acid amplification into one single chip, providing a good reference for developing POCT with sample-in-answer-out capability.

## Conclusions

Given the high population density, fast pace of life, and high mobility of people in today’s world, a high risk of a global pandemic caused by the outbreak of newly emerging highly infectious diseases exists. The practice of COVID-19 prevention and control has demonstrated that the rapid diagnosis of infectious diseases is an important link for the epidemic control of diseases in a timely manner. Early detection, early diagnosis, and early quarantine can effectively control the epidemic spread. It is of great practical significance for epidemic prevention and control to develop rapid pathogen detection techniques applicable to the outbreak sites and remote grassroots. POCT with combined advantages of isothermal amplification and microfluidic chip technology possesses the advantages of short assay time, simple operation, compactness and portability, low diagnostic cost, and the avoidance of aerosol contamination in the rapid on-site detection of pathogens, thus, showing a remarkable application prospect. However, its wide application in on-site and clinical detection faces the following challenges: (1) it is unaffordable for ordinary laboratories because of a high research cost, complex chip fabrication procedure, and time-consuming and labor-intensive preparation and fixation of primers and probes; (2) it has poor comparability of various data and difficulty in sharing the data due to the differences in the equipment and sample processing methods among different laboratories; (3) no effective and unified standards are currently available for sample preparation and detection, as well as data processing, thereby significantly influencing the reproducibility of chip detection results; (4) compared with laboratory testing, POCT testing is subject to more influencing factors and gives rise to less stable results; (5) microfluidic chips are mostly disposable and produce a lot of medical and sanitary wastes. As a result, the later recycling remains a difficult issue; and (6) the integration and automated operation of multiple processing links involved in pathogen detection on the microfluidic chip has not yet been achieved. In this case, manual operations such as nucleic acid extraction and purification are still required. Hence, the aforementioned issues need to be addressed in the future. Specifically, future work should be directed at further optimizing and improving the compatibility of isothermal amplification combined with the microfluidic chip technique, promoting the detection performance indicators, and reducing the production cost. Moreover, relevant technical standards need to be formulated to improve the quality control of detection activities as well as the reliability and comparability of the test results. It is believed that POCT will play a more important role in pathogen detection and disease diagnosis in the real world with continuous development and improvement of microfluidic chip technology, miniaturization, and reduced cost of detection instruments, as well as the growing maturity of industrialization.

## Data Availability

Not applicable.

## Abbreviations

COVID-19: Coronavirus disease 2019
PCR: Polymerase chain reaction
qPCR: Quantitative polymerase chain reaction
POCT: Point-of-care testing
RPA: Recombinant polymerase isothermal amplification
SARS-CoV-2: Severe acute respiratory syndrome coronavirus
qRT-PCR: Quantitative real-time fluorescent PCR
mμLAMP: Multiplex microfluidic system based on loop-mediated isothermal amplification
ESKAPE: *Enterococcus faecium*, *S. aureus*, *Klebsiella pneumoniae*, *Acinetobacter baumannii*, *Pseudomonas aeruginosa*, and *Enterobacter spp*
PDMS: Polydimethylsiloxane
i-centrifuge: Inertial microfluidic centrifuge
LAMP: Loop-mediated isothermal amplification
RCA: Rolling circle amplification
HAD: Helicase-dependent amplification
LFD: Lateral flow dipstick
LF: Lateral flow
THF: Tetrahydrofuran
RT-RPA: Reverse-transcription recombinant polymerase isothermal amplification
DMF: Digital microfluidics
MI-IF-RPA: Microfluidic-integrated lateral flow recombinase polymerase amplification
TB: Tuberculosis
Fd-MC: Finger-driven microfluidic system

## References

[1] Nii-Trebi NI (2017). Emerging and Neglected Infectious Diseases: Insights, Advances, and Challenges. Biomed Res Int..

[2] Hao R, Liu Y, Shen W, Zhao R, Jiang B, Song H (2022). Surveillance of emerging infectious diseases for biosecurity. Sci China Life Sci..

[3] Liu Q, Cao L, Zhu XQ (2014). Major emerging and re-emerging zoonoses in China: a matter of global health and socioeconomic development for 1.3 billion. Int J Infect Dis..

[4] Reported Cases and Deaths by Country or Territory. www.worldometers.info/coronavirus/. Accessed 20 May 2022.

[5] Sun J, Xianyu Y, Jiang X (2014). Point-of-care biochemical assays using gold nanoparticle-implemented microfluidics. Chem Soc Rev..

[6] Muralidar S, Ambi SV, Sekaran S, Krishnan UM (2020). The emergence of COVID-19 as a global pandemic: Understanding the epidemiology, immune response and potential therapeutic targets of SARS-CoV-2. Biochimie..

[7] Adhikari SP, Meng S, Wu YJ, Mao YP, Ye RX, Wang QZ (2020). Epidemiology, causes, clinical manifestation and diagnosis, prevention and control of coronavirus disease (COVID-19) during the early outbreak period: a scoping review. Infect Dis Poverty..

[8] Mabey D, Peeling RW, Ustianowski A, Perkins MD (2004). Diagnostics for the developing world. Nat Rev Microbiol..

[9] Mullis KB, Faloona FA (1987). Specific synthesis of DNA in vitro via a polymerase-catalyzed chain reaction. Methods Enzymol..

[10] Saingam P, Li B, Yan T (2018). Use of amplicon sequencing to improve sensitivity in PCR-based detection of microbial pathogen in environmental samples. J Microbiol Methods..

[11] Wang M, Yang J, Gai Z, Huo S, Zhu J, Li J (2018). Comparison between digital PCR and real-time PCR in detection of Salmonella typhimurium in milk. Int J Food Microbiol..

[12] Cao Y, Yu M, Dong G, Chen B, Zhang B. Digital PCR as an Emerging Tool for Monitoring of Microbial Biodegradation. Molecules. 2020;25(3). 10.3390/molecules25030706 PMC7037809 32041334

[13] Notice on Issuing the Novel Coronavirus Antigen Testing Application Plan. http://www.gov.cn/xinwen/2022-03/11/content_5678610.htm. Accessed 20 May 2022.

[14] Abel G (2015). Current status and future prospects of point-of-care testing around the globe. Expert Rev Mol Diagn..

[15] Liao RJ, Ji-Ke CN, Zhang T, Liao Q, Li L, Zhu TY (2020). Coronavirus disease 2019 epidemic in impoverished area: Liangshan Yi autonomous prefecture as an example. Infect Dis Poverty..

[16] Florkowski C, Don-Wauchope A, Gimenez N, Rodriguez-Capote K, Wils J, Zemlin A (2017). Point-of-care testing (POCT) and evidence-based laboratory medicine (EBLM) - does it leverage any advantage in clinical decision making?. Crit Rev Clin Lab Sci..

[17] Wiencek J, Nichols J (2016). Issues in the practical implementation of POCT: overcoming challenges. Expert Rev Mol Diagn..

[18] Ferreira CES, Guerra JCC, Slhessarenko N, Scartezini M, Franca CN, Colombini MP (2018). Point-of-Care Testing: General Aspects. Clin Lab..

[19] Linshiz G, Jensen E, Stawski N, Bi C, Elsbree N, Jiao H (2016). End-to-end automated microfluidic platform for synthetic biology: from design to functional analysis. J Biol Eng..

[20] Jayamohan H, Sant HJ, Gale BK (2013). Applications of microfluidics for molecular diagnostics. Methods Mol Biol..

[21] He S, Joseph N, Feng S, Jellicoe M, Raston CL (2020). Application of microfluidic technology in food processing. Food Funct..

[22] Zhang D, Bi H, Liu B, Qiao L (2018). Detection of Pathogenic Microorganisms by Microfluidics Based Analytical Methods. Anal Chem..

[23] Park J, Han DH, Park JK (2020). Towards practical sample preparation in point-of-care testing: user-friendly microfluidic devices. Lab Chip..

[24] Xu L, Wang A, Li X, Oh KW (2020). Passive micropumping in microfluidics for point-of-care testing. Biomicrofluidics..

[25] Sin MLY, Gao J, Liao JC, Wong PK (2011). System Integration - A Major Step toward Lab on a Chip. J Biol Eng..

[26] Liu D, Shen H, Zhang Y, Shen D, Zhu M, Song Y (2021). A microfluidic-integrated lateral flow recombinase polymerase amplification (MI-IF-RPA) assay for rapid COVID-19 detection. Lab Chip..

[27] Fang X, Chen H, Yu S, Jiang X, Kong J (2011). Predicting viruses accurately by a multiplex microfluidic loop-mediated isothermal amplification chip. Anal Chem..

[28] Renner LD, Zan J, Hu LI, Martinez M, Resto PJ, Siegel AC, et al. Detection of ESKAPE Bacterial Pathogens at the Point of Care Using Isothermal DNA-Based Assays in a Portable Degas-Actuated Microfluidic Diagnostic Assay Platform. Appl Environ Microbiol. 2017;83(4). 10.1128/AEM.02449-16 PMC5288812 27986722

[29] Wang A, Boroujeni SM, Schneider PJ, Christie LB, Mancuso KA, Andreadis ST, et al. An Integrated Centrifugal Degassed PDMS-Based Microfluidic Device for Serial Dilution. Micromachines (Basel). 2021;12(5). 10.3390/mi12050482 PMC8145514 33922553

[30] Chen P, Chen C, Su H, Zhou M, Li S, Du W, et al. Integrated and finger-actuated microfluidic chip for point-of-care testing of multiple pathogens. Talanta. 2021;224. 10.1016/j.talanta.2020.121844 33379062

[31] Xiang N, Ni Z. Hand-Powered Inertial Microfluidic Syringe-Tip Centrifuge. Biosensors. 2021;12:1. 10.3390/bios12010014 PMC8774109 35049644

[32] Notomi T, Okayama H, Masubuchi H, Yonekawa T, Watanabe K, Amino N (2000). Loop-mediated isothermal amplification of DNA. Nucleic Acids Res..

[33] Ravan H, Yazdanparast R (2012). Development and evaluation of a loop-mediated isothermal amplification method in conjunction with an enzyme-linked immunosorbent assay for specific detection of Salmonella serogroup D. Anal Chim Acta..

[34] Xue Y, Kong Q, Ding H, Xie C, Zheng B, Zhuo X (2021). A novel loop-mediated isothermal amplification-lateral-flow-dipstick (LAMP-LFD) device for rapid detection of Toxoplasma gondii in the blood of stray cats and dogs. Parasite..

[35] Nimitphak T, Kiatpathomchai W, Flegel TW (2008). Shrimp hepatopancreatic parvovirus detection by combining loop-mediated isothermal amplification with a lateral flow dipstick. J Virol Methods..

[36] Mori Y, Hirano T, Notomi T (2006). Sequence specific visual detection of LAMP reactions by addition of cationic polymers. BMC Biotechnol..

[37] Mori Y, Kitao M, Tomita N, Notomi T (2004). Real-time turbidimetry of LAMP reaction for quantifying template DNA. J Biochem Biophys Methods..

[38] Kashir J, Yaqinuddin A (2020). Loop mediated isothermal amplification (LAMP) assays as a rapid diagnostic for COVID-19. Med Hypotheses..

[39] Compton J (1991). Nucleic acid sequence-based amplification. Nature..

[40] Abdolahzadeh A, Dolgosheina EV, Unrau PJ (2019). RNA detection with high specificity and sensitivity using nested fluorogenic Mango NASBA. RNA (New York, NY)..

[41] Zeng W, Yao W, Wang Y, Li Y, Bermann SM, Ren Y (2017). Molecular detection of genotype II grass carp reovirus based on nucleic acid sequence-based amplification combined with enzyme-linked immunosorbent assay (NASBA-ELISA). J Virol Methods..

[42] Tillmann RL, Simon A, Muller A, Schildgen O (2007). Sensitive commercial NASBA assay for the detection of respiratory syncytial virus in clinical specimen. PLoS One..

[43] Deiman B, Schrover C, Moore C, Westmoreland D, van de Wiel P (2007). Rapid and highly sensitive qualitative real-time assay for detection of respiratory syncytial virus A and B using NASBA and molecular beacon technology. J Virol Methods..

[44] Jean J, Blais B, Darveau A, Fliss I (2002). Simultaneous detection and identification of hepatitis A virus and rotavirus by multiplex nucleic acid sequence-based amplification (NASBA) and microtiter plate hybridization system. J Virol Methods..

[45] Piepenburg O, Williams CH, Stemple DL, Armes NA (2006). DNA detection using recombination proteins. PLoS Biol..

[46] Fan X, Li L, Zhao Y, Liu Y, Liu C, Wang Q (2020). Clinical Validation of Two Recombinase-Based Isothermal Amplification Assays (RPA/RAA) for the Rapid Detection of African Swine Fever Virus. Front Microbiol..

[47] Nie Z, Zhao Y, Shu X, Li D, Ao Y, Li M (2021). Recombinase polymerase amplification with lateral flow strip for detecting Babesia microti infections. Parasitol Int..

[48] Li J, Macdonald J, von Stetten F (2018). Review: a comprehensive summary of a decade development of the recombinase polymerase amplification. Analyst..

[49] Banér J, Nilsson M, Mendel-Hartvig M, Landegren U (1998). Signal amplification of padlock probes by rolling circle replication. Nucleic acids research..

[50] Li Y, Wang J, Wang S, Wang J (2020). Rolling circle amplification based colorimetric determination of Staphylococcus aureus. Mikrochim Acta..

[51] Ciftci S, Neumann F, Abdurahman S, Appelberg KS, Mirazimi A, Nilsson M (2020). Digital Rolling Circle Amplification-Based Detection of Ebola and Other Tropical Viruses. J Mol Diagn..

[52] Zhang K, Zhang H, Cao H, Jiang Y, Mao K, Yang Z. Rolling Circle Amplification as an Efficient Analytical Tool for Rapid Detection of Contaminants in Aqueous Environments. Biosensors. 2021;11(10). 10.3390/bios11100352 PMC8533700 34677308

[53] Vincent M, Xu Y, Kong H (2004). Helicase-dependent isothermal DNA amplification. EMBO Rep..

[54] Zhao Y, Chen F, Li Q, Wang L, Fan C (2015). Isothermal Amplification of Nucleic Acids. Chem Rev..

[55] Zhou Q, Liu Y, Wang Z, Wang H, Zhang X, Lu Q (2022). Rapid On-Site Detection of the Bursaphelenchus xylophilus Using Recombinase Polymerase Amplification Combined With Lateral Flow Dipstick That Eliminates Interference From Primer-Dependent Artifacts. Front Plant Sci..

[56] Zhao G, Hou P, Huan Y, He C, Wang H, He H (2018). Development of a recombinase polymerase amplification combined with a lateral flow dipstick assay for rapid detection of the Mycoplasma bovis. BMC Vet Res..

[57] Gupta SK, Deng Q, Gupta TB, Maclean P, Jores J, Heiser A (2021). Recombinase polymerase amplification assay combined with a dipstick-readout for rapid detection of Mycoplasma ovipneumoniae infections. PLoS One..

[58] Kim TH, Park J, Kim CJ, Cho YK (2014). Fully integrated lab-on-a-disc for nucleic acid analysis of food-borne pathogens. Anal Chem..

[59] Choi G, Jung JH, Park BH, Oh SJ, Seo JH, Choi JS (2016). A centrifugal direct recombinase polymerase amplification (direct-RPA) microdevice for multiplex and real-time identification of food poisoning bacteria. Lab Chip..

[60] Huang Q, Shan X, Cao R, Jin X, Lin X, He Q, et al. Microfluidic Chip with Two-Stage Isothermal Amplification Method for Highly Sensitive Parallel Detection of SARS-CoV-2 and Measles Virus. Micromachines (Basel). 2021;12(12). 10.3390/mi12121582 PMC8705924 34945432

[61] Yang B, Kong J, Fang X (2019). Bandage-like wearable flexible microfluidic recombinase polymerase amplification sensor for the rapid visual detection of nucleic acids. Talanta..

[62] Kong M, Li Z, Wu J, Hu J, Sheng Y, Wu D (2019). A wearable microfluidic device for rapid detection of HIV-1 DNA using recombinase polymerase amplification. Talanta..

[63] Sun Z, Lin KF, Zhao ZH, Wang Y, Hong XX, Guo JG (2022). An automated nucleic acid detection platform using digital microfluidics with an optimized Cas12a system. Sci China Chem..

[64] Wang Z, Wang Y, Lin L, Wu T, Zhao Z, Ying B (2022). A finger-driven disposable micro-platform based on isothermal amplification for the application of multiplexed and point-of-care diagnosis of tuberculosis. Biosens Bioelectron..

